# Close relationships in Parkinson´s disease patients with device‐aided therapy

**DOI:** 10.1002/brb3.2102

**Published:** 2021-05-05

**Authors:** Monica Scharfenort, Jonathan Timpka, Thomas Sahlström, Tove Henriksen, Dag Nyholm, Per Odin

**Affiliations:** ^1^ Division of Neurology Lund University Lund Sweden; ^2^ Department of Neurology Skåne University Hospital Lund Sweden; ^3^ Department of Neurology University Hospital of Bispebjerg Copenhagen Denmark; ^4^ Department of Neuroscience Uppsala University Uppsala Sweden

**Keywords:** advanced Parkinson´s disease, apomorphine infusion, attachment theory, deep brain stimulation, device‐aided treatment, levodopa infusion, relationship satisfaction

## Abstract

**Objectives:**

Deep brain stimulation, continuous subcutaneous apomorphine infusion, and levodopa–carbidopa intestinal gel infusion, together called device‐aided therapies (DAT), are introduced when oral and transdermal pharmacotherapy are not enough for a satisfactory control of Parkinson's disease (PD) symptoms. Solid relationships are central to an individual's well‐being, but the impact of close relationships in advanced PD remains underexplored. The aim of this study was to investigate the development of close relationships between PD patients and their partners following the initiation of DAT and to examine the relationship structures in these relationships.

**Materials and Methods:**

This was a retrospective quantitative multicenter pilot study wherein 41 couples, patients with advanced PD and their partners, retrospectively rated their relationship satisfaction before the start of DAT, after one year of DAT and at the time of the interview. The couples also answered the Experiences in Close Relationships—Questionnaire of Relational Structures (ECR‐RS).

**Results:**

Partners more often report changes in relationship satisfaction than patients between baseline and both 1 year after start of DAT (*p* = .049) and last evaluation (*p* = .041). The ECR‐RS data reported significantly higher avoidance score for partners (*p* = .005) and significantly higher anxiety score for patients (*p* = .024).

**Conclusions:**

The close relationship wherein one part has PD and receives DAT has a high risk of being unequal. Prospective studies are needed for further clarification of the interplay between advanced PD, DAT, and close relationships, this in order to improve pre‐ and postinterventional support for PD patients receiving DAT, as well as their partners.

## Graphical abstract

The close relationship wherein one part has PD and receives Device‐Aided Therapy has a high risk of being unequal. Partners more often report changes in relationship satisfaction and report significantly higher avoidance score on the ECR‐RS in contrast to patients who reports significantly higher anxiety scores.

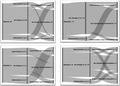

## 1 INTRODUCTION

In addition to the characteristic combination of motor symptoms, Parkinson's disease (PD) results in a wide range of nonmotor symptoms; emerging from nearly every organ system and causes cognitive impairment and sleep disorders, sexual dysfunction, gastrointestinal problems, or anosmia, among other symptoms (Bhat et al., 2018). PD is a progressive disease, and complications that affect aspects of daily functioning increase the patient's dependency on caregiving and support over time. Informal caregivers, such as family members, are often a primary source of support. It is well known that both nonmotor and motor symptoms affect patients’ and family caregivers’ quality of life (QoL) (Hurt et al., 2017). According to the World Health Organization (WHO) International Classification of Functioning, Disability and Health (ICF), health is described as *Body function*, *Body structure*, *Activity and participation* and *Environmental factors* (World Health Organization, 2002). Environment issues, including the living situation, family and friends, have gained interest lately and more is to learn in order to find factors of importance for the well‐being of patients and family/partners (Van Uem et al., 2016; World Health Organization, 2002). The progression of PD often leads to a disruption of plans and, subsequently, a need for stabilization of daily routines (Hurt et al., 2017; Lau & Au, 2011). There is a widespread trend toward fewer and shorter hospital stays and patients are, to a greater extent, left to self‐manage their illness at home (Sundström & Johansson, 2004). Family caregivers are thus of economic benefit for the society (Jorgensen et al., 2009), but the increasing responsibilities for family caregivers can lead to emotional, economic, and social strain (Hempel et al., 2008; Theed et al., 2017). The support provided to family caregivers is often lacking, despite an evident connection between the well‐being of the family caregiver and that of the patient (Hand et al., 2019). A limited amount of studies have examined the psychological and psychosocial impacts of PD on family caregivers. The existing literature often includes only one side, patient or family caregiver, and often without consideration of the ongoing therapy (Greenwell et al., 2015).

Device‐aided therapy (DAT), such as *deep brain stimulation* (DBS), *continuous subcutaneous apomorphine infusion* (CSAI), and *levodopa–carbidopa intestinal gel infusion* (LCIG), is introduced at a stage when patients have developed motor fluctuations. At this point in time, the PD symptoms have a significant negative impact on the activities of daily living, despite optimized oral and transdermal pharmacotherapy. A major effect of moving from conventional therapy to advanced therapy is that motor fluctuations are stabilized, with less time in “off”‐mode(?), less time with troublesome dyskinesias, but also with improvement of many nonmotors symptoms and health‐related QoL (Haidar S. Dafsari et al., 2019). The aim with DAT is to increase the patient's independence. But despite positive effects of DATs in a majority of patients, some studies have indicated that the help required shifts to other areas and caregiver burden thereby remains largely unchanged (Nyholm et al., 2012; Santos‐García et al., 2012; Soileau et al., 2014). There has been a recent increase in the interest in how PD affects relationships, but there is a remaining knowledge gap for close romantic relationships, despite the known importance of close relationships for the patient's QoL (Hodgson et al., 2004; Karlstedt et al., 2018; Tanji et al., 2008; Theed et al., 2017). The same tendency is evident in the limited number of studies including partners of PD patients undergoing DAT (Lewis, Maier, Horstkötter, Eggers, et al., 2015; Soileau et al., 2014).

The attachment theory is often employed due to its explanatory power and clear relevance for health‐related behavior and outcome (Mikulincer & Shaver, 2005; Pietromonaco et al., 2013). Individual differences in attachment style are believed to shape the individuals’ health behavior and outcome and are believed to predict whether and how an individual seeks support from a close partner as well as their ability to provide comfort and reassurance when the partner is in need (Feeney & Collins, 2015; Pietromonaco et al., 2013). For example, in the face of declining health the attachment styles influences if the individual seek out an attachment figure (often partner) in an attempt to restore emotional well‐being. In the same way, the partners respond according to their attachment style, for example, by providing care through reassurance, comfort, and support. The *Experiences in Close Relationships—Questionnaire of Relational Structures* (ECR‐RS) evaluates and contextualizes the assessment of attachment styles in adult relationships. In contrast to other attachment questionnaires, the ECR‐RS specifies the assessed relationship, *best friend*, *romantic partner*, *mother*, *father,* or *parental figure*, and it allows for intrapersonal variations across a relational context (Fraley et al., 2011). It is also possible to only target one or some of the domains, for instance, only the best friend and parent as seen in Feddern et al (Feddern Donbaek & Elklit, 2014). The ECR‐RS scores two different dimensions: *avoidance* and *anxiety* (Rocha et al., 2017). *Attachment avoidance* characterizes the individual's discomfort of intimacy and refers to the strive for independency and the perception of their partner's real intentions, for example, “I don´t feel comfortable opening up to this person” versus “I find it easy to open up to this person.” *Attachment anxiety* characterizes the individual's fear of abandonment and rejection and refers to the individual's perception of the partner's estimated ability to support and their availability in time of need, for example, “I´m afraid this person will abandon me.” In a secure attachment, both the anxiety and avoidance dimensions are low (Fraley et al., 2011; Lafontaine et al., 2016).

The aim of this study was to investigate the close relationships between PD patients and their partners after the initiation of DAT. The research questions were (a) if the patients satisfaction with their close relationship changes before or after the initiation of DAT, (b) if the partners of the PD patients satisfaction with their close relationships changes during the same period, and (c) how do the attachment structures differ in the close relationship between PD patients receiving DAT and their partners?

## 2 MATERIALS AND METHOD

This was a retrospective quantitative pilot study including patients identified through the Swedish National Parkinson Patient Registry, ParkReg (a part of Swedish Neuro Registries) or by the treating neurologist at three Movement Disorder Centers: two in Sweden (Skåne University Hospital and Uppsala University Hospital) and one in Denmark (Bispebjerg University Hospital). The inclusion criteria for patients were a diagnosis of idiopathic PD, an age younger than 67 years at the initiation of DAT, and a total therapy duration with one of the DAT for a minimum of 1 year. Exclusion criteria were, among both patients and partners, severe cognitive impairment or dysarthria that affected the ability to answer the interview questions. Patients with a history of more than one DAT were categorized by their first device‐aided therapy if the duration of that therapy was more than 1 year. Eligible patients and their spouses received written information about the study and signed a written consent before participating in the telephone interview. The Regional Ethical Review Board in Lund, Sweden, approved the study (project‐id 2017/635).

The ECR‐RS questionnaire is a nine‐item, self‐reported measurement that captures two dimensions: attachment‐related avoidance (item 1–6) and anxiety (item 7–9) in close relationships (Fraley et al., 2011). All nine items have a 7‐point Likert scale that ranges from 1 (strongly disagree) to 7 (strongly agree). The individual's attachment‐related anxiety is the mean of item 7–9, and the avoidance is the reverse key mean of item 1–6. A low score gives a lower anxiety or avoidance‐related attachment. The global attachment‐related anxiety or avoidance is the mean anxiety or avoidance of all four domains: a mean score of the relationship with mother, father, close friend, and romantic partner. Only the romantic partner was targeted in the present study.

Participants stated their relationship status retrospectively for two points in time: baseline (before the initiation of DAT) and after one year of DAT, but also the relationship status at the time of the interview (i.e., the last evaluation). Furthermore, they assessed their relationship satisfaction in a similar manner, using a Likert scale from 1 (very dissatisfied) to 7 (very satisfied).

### 2.1 Statistics

Nonparametric statistical methods were used. Statistical differences were evaluated using the Wilcoxon signed‐rank test, Pearson's chi‐square *t* test, or Kruskal–Wallis test. IBM SPSS Statistics for Windows (version 25, **RRID** **:S** **CR_019096**) was used for statistical analyses. *p* ≤ .05 was considered significant.

## 3 RESULTS

Out of the 114 contacted patients, 67 patients (59%) and 41 partners agreed to participate and gave their consent. 26 patients were excluded from the rating of relationship satisfaction assessment, either due to the spouse declining to participate (eight patients), the patient being unwilling to let the spouse participate (one patient) or being single at the last evaluation (17 patients). Out of the 41 included couples (Table 1), two underwent a change in relationship status: one went from being single both before and 1 year after start of DBS to living together at the last evaluation and the other couple, treated with CSAI, went from cohabitant to getting married. Because of missing data on relationship satisfaction, only 8 CSAI partners were included in the statistical calculation in this question (Table 1).

**TABLE 1:** Description of participants and distribution between types of treatment

	Total	DBS	CSAI	LCIG
Couples included (female/male^a^)	41 (15/26)	17 (7/10)	10 (3/7)	14 (6/8)
Age, years	66 (47–73)	63 (47–70)	66.5 (51–72)	67 (50–73)
Disease duration, years	16 (6–31)	15 (9–28)	15.5 (6–24)	20.5 (12 – 31)
DAT^b^ duration, years	4 (2–13)	3 (2–13)	5.5 (2–12)	6.5 (3–13)
Time from diagnosis to DAT, years	10 (2–28)	10 (5–25)	9.5 (2–18)	12.5 (6–28)

^a^ Refers to the patients’ gender.

^b^ DAT, Device‐Aided Therapy, includes the three therapies: CSAI, continuous subcutaneous apomorphine infusion; DBS, deep brain stimulation; LCIG, levodopa–carbidopa intestinal gel. Data are shown as medians (range) unless otherwise noted.

**FIGURE 1:**
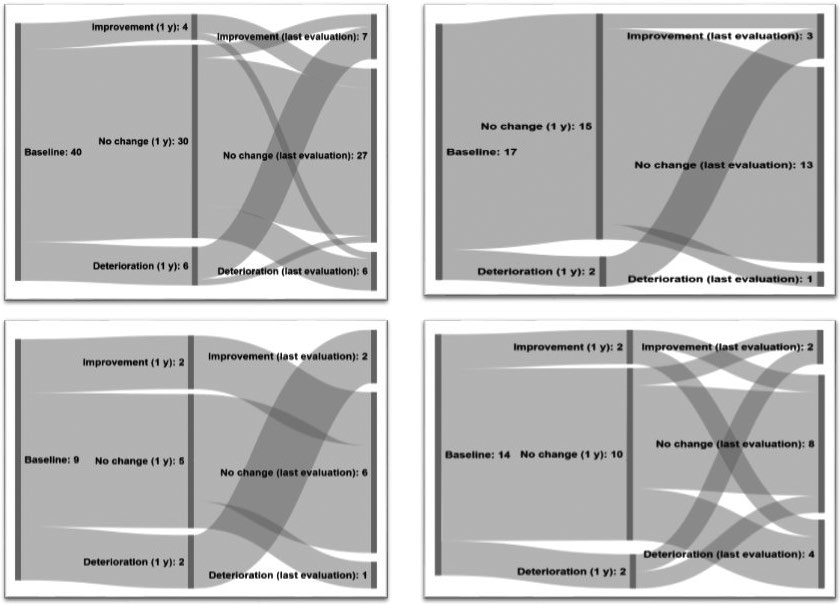
Changes in patients’ relationship satisfaction over time. The first node represents the baseline, the second node the change in relationship satisfaction one year after start of Device‐Aided Therapy compared to before start. The third node represents the change in relationship satisfaction at last evaluation compared to 1 year after start. a) patients with all the three different Device‐Aided Therapies (DAT), b) patients with Deep Brain Stimulation (DBS). c) patients with Continuous Subcutaneous Apomorphine Infusion (CSAI) and d) patients with Levidopa–carbidopa Intestinal Gel (LCIG)

**FIGURE 2:**
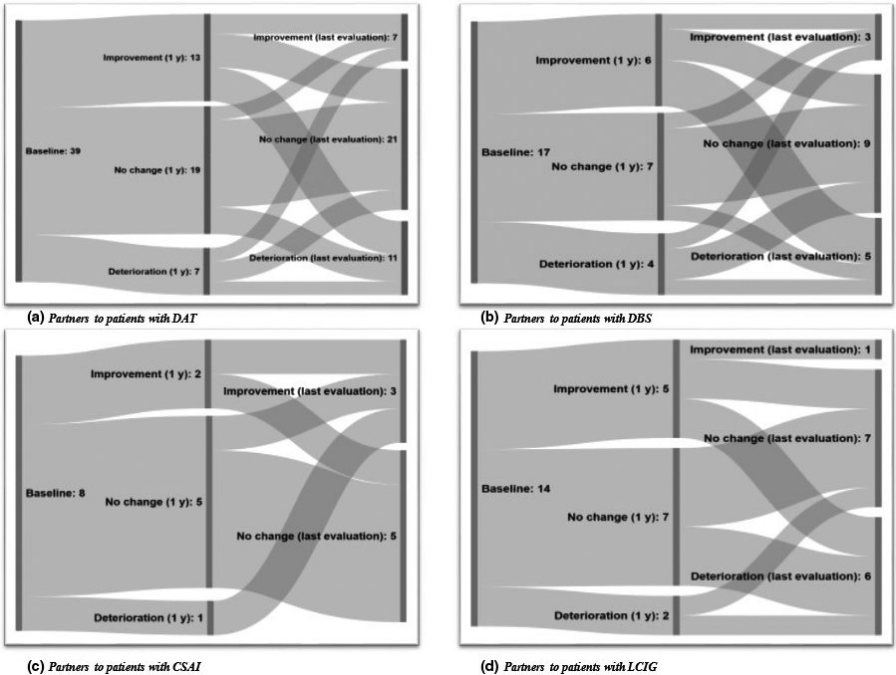
Changes in partners’ relationship satisfaction over time. The first node represents the baseline, the second node the change in relationship satisfaction 1 year after start of Device‐Aided Therapy compared to before start. The third node represents the change in relationship satisfaction at last evaluation compared to 1 year after start. a) partners to patients with all the three different Device‐Aided Therapies (DAT). b) partners to patients with Deep Brain Stimulation (DBS). c) partners to patients with Continuous Subcutaneous Apomorphine Infusion (CSAI) and d) partners to patients with Levidopa–carbidopa Intestinal Gel (LCIG)

Changes in the appreciation of the relationships as well as the direction of the change varied for both patients and partners over time (Figures 1 and 2), but no significant differences were found. When comparing patients and partners, the partners reported significantly more relationship satisfaction changes between baseline and both 1 year after start of DAT (*p* = .049) and last evaluation (*p* = .041; Table 2). In contrast, differences in relationship satisfaction changes between 1 year after start of DAT and last evaluation were not significant (*p* = .21). Neither was any significant difference found when dividing the data by gender, by the three therapy groups nor in relation to point of time.

**TABLE 2:** Changes in relationship satisfaction over time, divided by patient and partner

	Baseline to last evaluation		Baseline to 1 year after start of DAT		1 year after start of DAT to last evaluation	
	No change	Change	No change	Change	No change	Change
Patients	62%	38%	75%	25%	67%	33%
Partners	38%	62%	50%	50%	51%	49%
*p*	**0.049**		**0.041**		0.210	

Note

Relationship satisfaction at baseline compared to last evaluation, baseline compared to after 1 year of DAT and after 1 year of DAT compared to at last evaluation. “Change” is either an increased or decreased relationship satisfaction.

DAT = Device‐Aided Therapy, includes the three therapies: CSAI, continuous subcutaneous apomorphine infusion; DBS, deep brain stimulation; LCIG, levodopa–carbidopa intestinal gel.

ECR‐RS data were included from all the 41 couples. The total avoidance score was significantly higher for partners (*p* = .005), and patients had significantly higher anxiety for abandonment (*p* = .024; Table 3). There was a significant difference between patients and partners regarding avoidance in the LCIG group (*p* = .009). When comparing male patients with female patients, there were no significant differences for avoidance (*p* = .95) or anxiety (*p* = .46).

**TABLE 3:** ECR‐RS score for patients and their partners

	Patients	Partners	*p*
DBS, *N*=	17	17	
*ECR‐RS: AVOIDANCE* Median (min‐max)	2.17 (1–4)	2.6 (2–5)	*0.055*
*ECR‐RS: ANXIETY* Median (min‐max)	1.33 (1–4)	1 (1–3)	*0.501*
CSAI, *N*=	10	10	
*ECR‐RS: AVOIDANCE* Median (min‐max)	1.92 (1–5)	2.08 (1–4)	*1.000*
*ECR‐RS: ANXIETY* Median (min‐max)	3 (1–5)	1.33 (1–5)	*0.093*
LCIG, *N*=	14	14	
*ECR‐RS: AVOIDANCE* Median (min‐max)	2.17 (1–5)	2.42 (2–6)	*0.009*
*ECR‐RS: ANXIETY* Median (min‐max)	2.33 (1–5)	2.17 (1–4)	*0.241*
TOTAL DAT, *N*=	41	41	
*ECR‐RS: Avoidance* Median (min‐max)	2.00 (1–5)	2.33 (1–6)	*0.005*
*ECR‐RS: Anxiety* Median (min‐max)	2.00 (1–5)	1.33 (1–5)	*0.024*

Note

ECR‐RS, Experiences in Close Relationships—Questionnaire of Relational Structures; DBS, deep brain stimulation; CSAI, continuous subcutaneous apomorphine infusion; LCIG, levodopa–carbidopa intestinal gel; DAT, device‐aided therapy.

A high score of avoidance shows a higher strive for independency. A high score of anxiety shows a higher fear of being abandoned.

## 4 DISCUSSION

The understanding of how the initiation of a DAT in advanced PD affects the patient's close relationships is currently limited. The primary finding of this retrospective study, including both PD patients with a DAT and their partners, was that there was a significant difference in relationship satisfaction between patients and partners. Partners more often reported a change in relationship satisfaction after the initiation of DAT, but there was no clear direction of the change. Furthermore, we found significant differences in the attachment style between patients and partners: Partners tend to show more attachment‐related avoidance and patients more attachment‐related anxiety.

In our study, the most stable period in terms of perceived relationship quality is between 1 year after start of DAT and the last evaluation, approximately five years. This is in line with the clinical experience that the effect of DAT develops during the first 3–6 months and then stabilizes. It is likely that, because of the start of DAT, an increased support is gained, which may also have an impact on the relationship satisfaction. In comparison, the biggest perceived difference is between baseline and last evaluation. The partners reported changes in relationship satisfaction to a higher degree than patients. In the context of activity and social loss, a feeling of lost closeness may emerge due to the change in roles in the caregiver–patient dyad, often depending on the task of the day; for example, caregiver one minute and spouse the next(Martin, 2016). The difference in relationship satisfaction and anxiety attachment styles suggests that healthcare providers should also consider the partners’ attachment style and a holistic couple‐based intervention is therefore of interest. Individual differences in attachment style impact the interaction in close relationships, as well as the interaction with healthcare providers: For example, the physician's perception of a difficult patient (Maunder et al., 2006) and the patient's feeling of trust for their own physician (Hillen et al., 2014) are related to attachment styles. In other words, an insecure attachment style gives a more insecure relationship perception.

We found that patients with PD and DAT experience anxiety of being abandoned, while their partners wish for more independence and tend to avoid intimacy, especially partners to LCIG patients. The differences in attachment point to a greater discomfort of intimacy and a strive for independence among partners while patients feel a greater fear of abandonment and rejection. This study does not investigate whether this is a consequence of living in a relationship, wherein one of the parts has a chronic disease, whether this is intrinsic to PD, due to impaired cognition or whether it is related to the DAT. The impact of DAT on other symptoms than motor state has previously not been well‐studied (Haidar Salimi Dafsari et al., 2016; Lang et al., 2016; Oyama et al., 2014; Reddy et al., 2014), but the increasing focus on nonmotor symptoms has lately been showing results. With an increased understanding of the multidimensional symptoms associated with PD, the clinicians can better foresee the potential impact of the therapy and help the patient set up realistic goals and make active and informed choices (Martin, 2016; Reddy et al., 2014).

During the last decades, several studies have investigated chronically ill patients’ social, psychosocial, and relational context (Karlstedt et al., 2017; O’Connor & McCabe, 2011; World Health Organization, 2002) and found a connection between interpersonal factors and an impact on biological processes as well as caregiving and health (Lo et al., 2009). There is a discrepancy between the significant impact of close relationships on the well‐being of patients and the low number of published studies (Van Uem et al., 2016). The ability to maintain continuity and a sense of normality is an important factor for QoL in both patients and partners. Impaired psychosocial functioning is strongly associated with negative health‐related QoL (Van Uem et al., 2016). Psychological problems in PD patients are often linked to the feeling of being a burden or the fear of being isolated; factors that are influencing and being influenced by close relationships (Hodgson et al., 2004). The interconnectedness of these issues shows the need for treatment, evaluation, and research in the three domains: biological, psychological, and social relationships, as described in WHO´s ICF (World Health Organization, 2002). This calls for a broadening of DAT impact beyond motor and nonmotor symptoms, including a more inclusive take on QoL and the context in which the patient lives. A more holistic approach is needed in times of personalized health care and support, including an assessment of both patients’ and partners’ needs (Hudson et al., 2010; Karlstedt et al., 2018; Reddy et al., 2014).

How a couple adjust to a chronic illness depends on a number of contextual factors, but chronic illness almost always changes relationships (Drutyte et al., 2014; Hodgson et al., 2004; Martin, 2016). Many chronic illnesses lead to loss of physical control energy and hope as well as independency, leading to lower self‐esteem and increased risk for depression. The influence of nonmotor symptoms on caregiver strain has previously been shown (Davis et al., 2011; Hand et al., 2019; Karlstedt et al., 2017), and changes in personality and mood in both positive and negative direction have been proposed in patients with DBS (Lewis, Maier, Horstkötter, Zywczok, et al., 2015). Depression and cognitive impairment are common nonmotor symptoms and have a significant impact on the health‐related QoL for both patient and partner (Chaudhuri et al., 2019; den Brok et al., 2015; Tessitore et al., 2018). A study on DBS reports a significant discrepancy in the perception of QoL between patient and partners where the patients report improved QoL while the partners report a decrease (Lewis et al., 2014). This may be ascribed to perceived behavior or mood change as well as challenges due to changes in the carer role (Liddle et al., 2018). The ability to adjust to health concerns is decreased if the patient is, as the results from present study reports, anxious and the partner avoidant (Lo et al., 2009; Pietromonaco et al., 2013), with severe symptoms possibly triggering the insecure attachment styles. Caregiver strain may increase in this case because of conflictual interactions and the couple's attachment style is of interest (Martin, 2016; Pietromonaco et al., 2013).

On the group level, men and women differ in coping with chronic diseases in the context of their relationship. It is believed to reflect a biological and socialization process which lead to differences in coping with stressful events (Pietromonaco et al., 2013; Poyner‐Del Vento et al., 2018). Nyholm et al. discuss the findings in their follow‐up study of patients with LCIG where the females were overrepresented amongst the patient to drop out of DAT (Nyholm et al., 2012). There are likely several explanations to this, but it is possible that relationship structures could be a contributing factor. The present study showed no significant difference between male and female patients and we know, through the Sahlström et al. (Sahlström et al., 2018), that a majority of the included patients showed an unchanged or increased ability to perform activities after starting DAT, compatible with a positive impact on motor symptoms. However, there is a need for further research to determine the correlation between nonmotor symptoms, the attachment styles, the social context (including the dyadic process), and health outcomes.

The strength of this study is that it, in contrast to most prior studies on relationships in PD, includes both patients and partners as well as including patients using any of the three kinds of DAT. This study also has a number of limitations: Firstly, the design of the study makes it vulnerable to recall bias, as the retrospective assessment of relationship appreciation is very likely to be influenced by the current situation. Furthermore, only one relational target of the ECR‐RS was explored and patients without a partner were left out of the analysis. By including the population without partners, an analysis of attachment styles’ correlation to relationship status would be possible. A thing to consider is the quit large amount of dropout regarding partners and it is possible that the couples included are living in a more positive relationship and that a negative relationship would make couples less likely to consent to participate in the study. Lastly, by limiting the population to a patient age of under 67 years when introducing the DAT, the population may not be characteristic for patients with DAT who generally are older than the participants in this study.

This study elucidated the inequality in close relationships where one part has received DAT after being diagnosed with PD. Since this study cannot prove that the initiation of DAT itself is the contributing factor to the result, a prospective follow‐up study exploring more domains would be of interest. Identifying the most suitable DAT for a specific patient with advanced PD is still considered difficult. Future research needs to focus on which DAT that would result in the most optimal outcome to the individual patient. An RCT study would be of great value in order to compare the three treatments. An evaluation of the patient's social context has a place in an individualized pre‐interventional screening and is needed to optimize pre‐interventional information and postinterventional support to patients, partners, and family caregivers.

## 5 CONCLUSION

The close relationship wherein one part has PD and receives DAT has a high risk of being unequal. The effect of DAT on relationships and the effect of these therapies on the burden and quality of life of relatives are examples of aspects of these therapies that are relatively unexplored. Prospective studies are needed for further clarification of the interplay between advanced PD, DAT, and close relationships, this in order to improve pre‐ and postinterventional support for PD patients receiving DAT, as well as their partners.

## CONFLICTS OF INTEREST

MS has served as a consultant to AbbVie and TLV. JT has served as a consultant to AbbVie. PO has received lecture fees and/or advisory board fees from AbbVie, NordicInfu Care, Stada, Britannia, and Lobsor. TH has received lecture fees from AbbVie, EVER Pharma, Nordic Infucare, and Britannia. DN has received lecture fees from AbbVie and NordicInfu Care. TS has reported no conflict of interest.

## AUTHOR CONTRIBUTION

MS, JT, and TS contributed to conception and design, acquisition, analysis and interpretation of data, participant recruitment, drafting and revising manuscript and final approval. TH and DN contributed to conception and design, participant recruitment, content expertise, revising it critically for important intellectual content and final approval. PO contributed to concept and design, interpretation, acquisition, and analysis of data, revising it critically for important intellectual content and final approval.

### PEER REVIEW

The peer review history for this article is available at https://publons.com/publon/10.1002/brb3.2102.
